# New records of nudibranchs and a cephalaspid from Kuwait, northwestern Arabian Gulf (Mollusca, Heterobranchia)

**DOI:** 10.3897/zookeys.1048.66250

**Published:** 2021-07-13

**Authors:** Manickam Nithyanandan, Manal Al-Kandari, Gopikrishna Mantha

**Affiliations:** 1 Ecosystem Based Management of Marine Resources, Environment and Life Sciences Research Center, Kuwait Institute for Scientific Research, P.O. Box.1638, Salmiya 22017, Kuwait Environment and Life Sciences Research Center, Kuwait Institute for Scientific Research Salmiya Kuwait

**Keywords:** Nudibranchs, diving, intertidal, Kuwait, Arabian Gulf

## Abstract

In this study five new records and two probably undescribed species of heterobranch sea slugs placed in four genera, three families, and two orders are reported from Kuwait, northwestern Arabian / Persian Gulf with details and photographs. The present study increases the heterobranch diversity in Kuwaiti waters from 35 to 40 species. The range of habitats in Kuwait provides a vital opportunity for further investigation to understand the actual faunal diversity.

## Introduction

Heterobranch sea slugs are one of the most colourful marine invertebrates, usually devoid of shells but in a few species, it is found externally and internally (e.g., Cephalaspidea, Aplysiida, Pleurobranchida), occurring in reefs, rocky habitats, and soft substrata (Yonow 2008; Mehrotra et al. 2021). The Arabian / Persian Gulf (APG) is a shallow marginal sea with a very wide range of temperature (10–48 °C) and salinity (42–65‰) and also highly impacted by anthropogenic activities (Sheppard et al. 2010). Kuwait lies in the northwestern APG, receiving freshwater input from Shatt-Al-Arab in Iraq, and has diverse habitats such as mud flats, sandy beaches, rocky shores, salt marshes, seagrass meadows, and coral reefs (Al-Yamani et al. 2004). The marine biodiversity of these productive habitats is unique and adapted to live in these extreme environmental conditions (salinity > 41 ppt and sea water temperature, 14 to > 30 °C) which falls beyond the physiological threshold for many organisms found elsewhere (Edmonds et al. 2021). Anthropogenic activities such as coastal development, pollution, etc. has immense impact on the fauna and flora in this marginal environment (Sheppard et al. 2010; Burt 2014).

The heterobranch fauna of APG are rather poorly documented with sporadic reports from Kuwait, Saudi Arabia, United Arab Emirates (UAE), and Iran (Glayzer et al. 1984; Jones 1986; Gosliner and Behrens 2004; Al-Yamani et al. 2012; Nithyanandan 2012; Yonow 2012; Gosliner et al. 2015; Rezai et al. 2016; Al-Kandari et al. 2020; Amini-Yekta and Dekker 2021). In Kuwaiti waters to date, 35 species of heterobranchs were recorded belonging to eighteen families and two orders (Al-Yamani et al. 2012; Nithyanandan 2012). In the present study new records of heterobranchs are documented from an offshore island and artificial marine habitats in Kuwaiti waters during the years 2012–2014.

## Materials and methods

**Figure 1.**
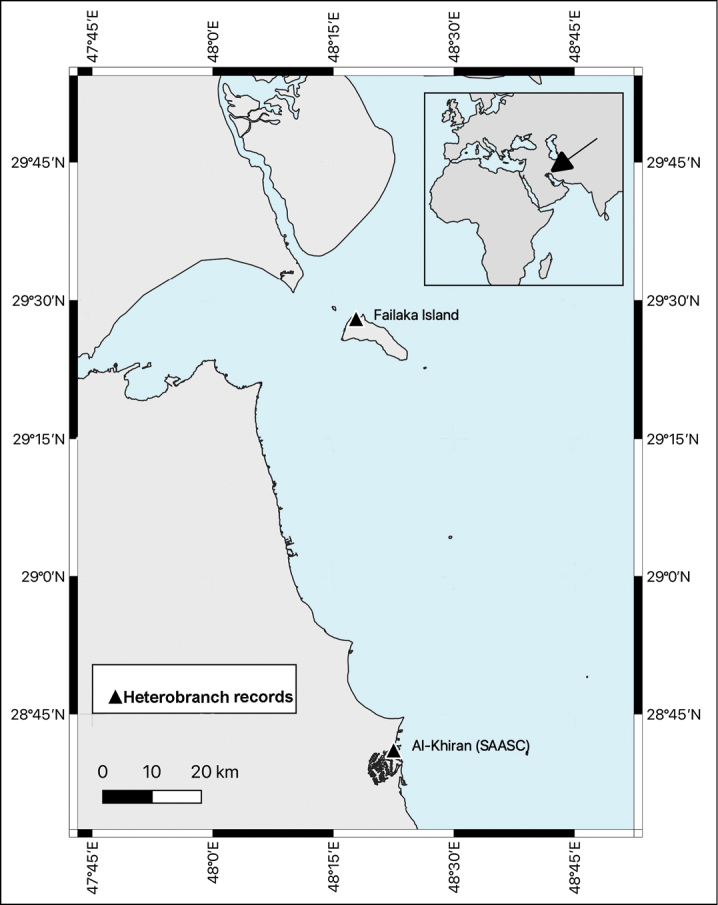
Map showing heterobranch record sites in Kuwait.

The Sabah Al-Ahmad Sea City (**SAASC**) is the largest coastal township development in Kuwait (Jones and Nithyanandan 2012a) covering an area up to 70 km^2^, with a network of artificial lagoons and habitats ranging from intertidal to subtidal zone (ca. 10 m depth). Heterobranchs were recorded from various artificial marine habitats (rock culverts, bridge piers, etc.) of SAASC (Fig. 1) by SCUBA diving while conducting routine underwater surveys for benthic monitoring during the years 2012–2014. Heterobranchs were photographed at 3–5 m depth using a digital camera (Panasonic LUMIX DMC-TZ7) with a waterproof casing. Due to the low density of animals observed, no attempts were made to collect reference specimens. No live measurements of the individuals were carried out. One individual was photographed from the rocky intertidal habitats of Failaka island during an extensive intertidal benthic survey in the winter of 2014. All morphological features described in this study are based on detailed examination of numerous photographs using Adobe Photoshop CS6. The classification adopted in this study is based on Bouchet et al. (2017) and, for nomenclature, the World Registry of Marine Species (WoRMS 2021) was followed. Identification of recorded individuals were based on Yonow (2008), Gosliner et al. (2015), and recent literature listed in WoRMS (2021).

## Taxonomic account

### Clade TECTIPLEURA Schrödl, Jörger, Klussmann-Kolb & Wilson, 2011

**Super Order EUOPISTHOBRANCHIA Jörger, Stöger, Kano, Fukuda, Knebelsberger & Schrödl, 2010**

**Order CEPHALASPIDEA P. Fischer, 1883**

**Family AGLAJIDAE Pilsbry, 1895 (1847)**

#### Genus *Chelidonura* A. Adams, 1850

##### Chelidonura livida

Taxon classificationAnimaliaCephalaspideaAglajidae

Yonow, 1994

4DBD274A-D37E-5D2D-B79F-AD6F199504E9

Figure 2

Aglaja cyanea (*nigra*): Engel and van Eeken 1962 (part): 17, E55/342 (Red Sea).
Chelidonura africana : Yonow 1990: 289, pl. 4 (Red Sea; misidentification).
Chelidonura livida Yonow, 1994a: 141–147, Fig. 1 (Eilat, Red Sea): Yonow 2008: 78–79, includes five figures (Gulf of Eilat, Red Sea).

###### Photographic record.

SAASC Al-Khiran, 13 June 2012, one individual photographed at 3 m depth in sandy substrate, R. Dinesh Kumar.

###### Description.

**Figure 2.**
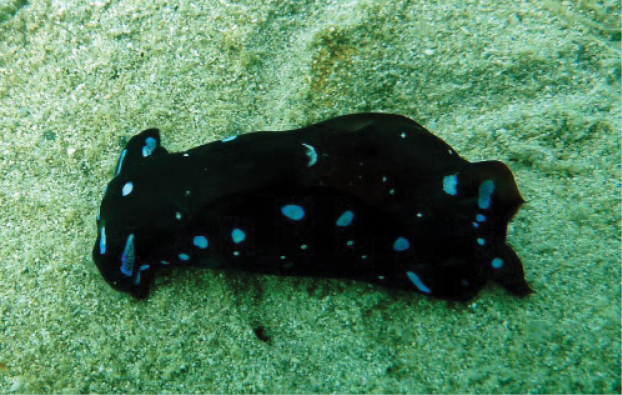
*Chelidonura livida* Yonow, 1994. Photograph R. Dinesh Kumar.

The individual has a black body colour, prominent electric blue spots scattered over the dorsum, head, and parapodia (Fig. 2). White flecks interspersed with electric blue spots are found on the head and along the edges of the parapodia. The caudal flaps are unequal with the left longer than the right, and with an electric blue spot at the base of the left caudal flap (see Fig. 2). The blue spots on the anterior portion of the propodium form a coalescent line which is partly visible in this individual (Fig. 2).

###### Distribution.

Israel (Yonow 1994a, 2008), Abu Dhabi (Hardy 2001), Mayotte Island (http://seaslugs.free.fr/nudibranche/a_cheli_livida.htm), Tanzania and Mozambique (Gosliner et al. 2008; Tibiriçá and Malaquias 2017), and Kuwait (this study).

###### Remarks.

Yonow (1994a) described *Ch. livida* from Eilat, Israel, in the north-eastern Red Sea. In *Ch. livida*, both sides of the mouth bear whitish or yellowish sensory bristles which is visible in the frontal view or if viewed from above (Yonow 1994a); however, it is not clearly visible in the photograph of the individual presented in this study due to the angle at which it was photographed (Fig. 2). The head shield has two short processes on its lateral side, which is bit longer in the left compared to the right side and tubular when the animal is in relaxed state (Yonow 1994a). This was clearly observed in the individual recorded in this study (Fig. 2). The individual recorded from Mozambique (Tibiriçá and Malaquias 2017: fig. 2f) has prominent electric blue rings that are scattered over the dorsum and parapodial margin. The caudal flaps are rather thin, the right one short and the left one elongated with a prominent electric blue spot. However, the individual observed in this study has short and thick caudal flap with a thin, pointed tip and a blue spot at its base (Fig. 2). The species possesses a highly reduced internal shell. This is a new record to both Kuwait and the APG, this record denoting a range extension into the northern APG from its type locality in the Red Sea.

#### Clade Nudipleura Wägele & Willan, 2000

**Order Nudibranchia Cuvier, 1817**

**Family Chromodorididae Bergh, 1891**

##### Genus *Goniobranchus* Pease, 1866

###### Goniobranchus bombayanus

Taxon classificationAnimaliaNudibranchiaChromodorididae

(Winckworth, 1946)

F6A494A5-8ED8-507B-BBBB-BB0C0C46E55F

Figure 3

Glossodoris bombayana Winckworth, 1946: 155–156, fig. 1 (Bombay, India).
Goniobranchus naiki Valdés, Mollo & Ortea, 1999: 468–471, fig. 1 (Mandapam, southern India); Gosliner et al. 2015: 228, one figure.

####### Photographic record.

SAASC, Al-Khiran, 23 March 2013, one individual photographed at 5 m depth on a concrete wall adjacent to tidal gates, Don Christopher Pereira.

####### Description.

**Figure 3.**
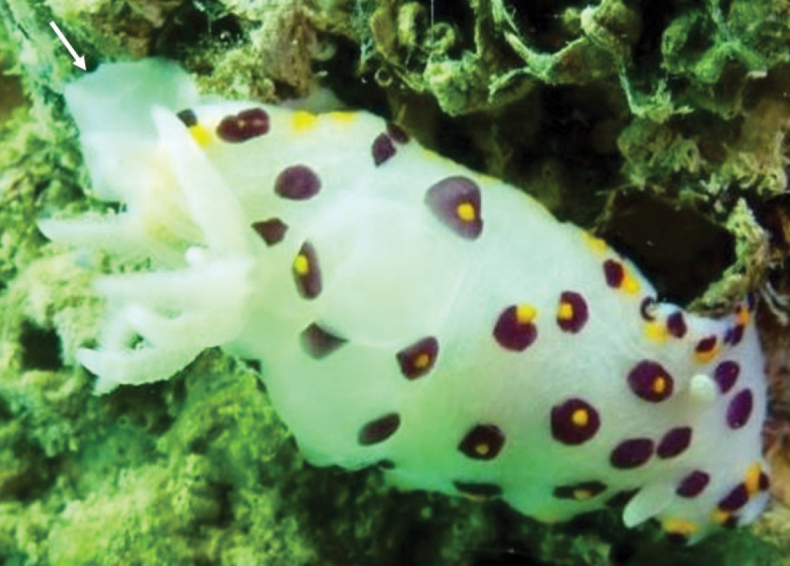
*Goniobranchus bombayanus* (Winckworth, 1946) (arrow indicates the white foot with no spots or markings). Photograph Don Christopher Pereira.

The individual photographed has a translucent white body with conspicuous deep purple spots scattered over the dorsum (Fig. 3). The foot is covered by the dorsum. On the mantle margin, yellow spots are arranged in a row merging with the purple spots. These yellow spots appear as a tubercle projecting from the centre of few purple spot on the dorsum and are confluent with purple spots in the margin. Rhinophores and gills bear rows of faint opaque white spots.

####### Distribution.

Known only from Mandapam, southern India (Valdés et al. 1999), Mumbai and Gulf of Kutch, northwestern India (Winckworth 1946; Apte and Desai 2017), and Kuwait (this study).

####### Remarks.

Johnson and Gosliner (2012), in considering the monophyletic nature of the genus *Chromodoris*, suggested a revision in the classification by moving some Indo-Pacific chromodorids to the genus *Goniobranchus*. According to WoRMS (2021)*Goniobranchus naiki* Valdez, Mollo & Ortea, 1999 from Mandapam, southern India is a junior synonym of *G. bombayanus* (Winckworth, 1946). In *G. naiki*, Valdés et al. (1999) and Gosliner et al. (2015) indicated the occurrence of translucent white spots on the dorsum; in the individual recorded during this study only faint opaque spots were observed (Fig. 3). In *G. bombayanus* the posterior portion of the foot extends beyond the mantle as a white tail with no dark spots (Winckworth 1946), which is also visible in the photographed individual (Fig. 3, arrowed). A new record to Kuwait and the APG.

###### Goniobranchus

Taxon classificationAnimaliaNudibranchiaChromodorididae

sp. 1

B82F77FC-A0B0-5074-93EB-E4A49E34AF5A

Figure 4

####### Photographic record.

SAASC, Al-Khiran, 23 March 2013, one individual photographed at 3.5 m depth on a rock culvert, R. Dinesh Kumar.

####### Description.

**Figure 4.**
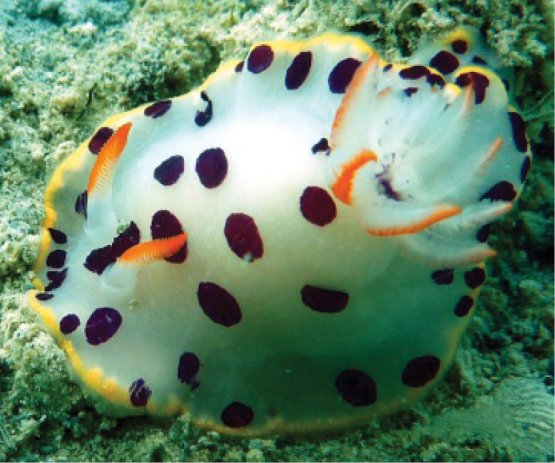
*Goniobranchus* sp. 1. Photograph R. Dinesh Kumar.

The individual has a white body with dark purple spots scattered over the dorsum and mantle margin (Fig. 4). A row of orange-yellow mantle glands covers the mantle margin. Rhinophores have a white base and bright orange lamellae gradually extending from the anterior surface up to 1/4 of the dorsal side, and the gills are white with a bright orange midrib.

####### Distribution.

Kuwait (this study) and Abu Dhabi, UAE Coast (http://medslugs.de/E/Ind-NW/Goniobranchus_sp_10/Goniobranchus_sp_10_01.htm)

####### Remarks.

Very similar to *G. kitae* (Gosliner 1994; see below) and *G. tumuliferus* (Collingwood, 1881) (see Gosliner et al. 2015 and Mehrotra et al. 2021). However, the individual observed in this study differs from both *G. kitae* and *G. tumuliferus* by having orange rhinophores with a white base, an orange midrib in the gills, purple spots on the elongated foot, and yellow at the tip of the elongated foot (Fig. 4). Probably an undescribed species.

###### Goniobranchus

Taxon classificationAnimaliaNudibranchiaChromodorididae

sp. 2

516F5290-8108-5F2F-902B-38B253DB9663

Figure 5

####### Photographic record.

Failaka Island, 22 December 2014, one individual found in rocks in sandy mud intertidal areas at the lowest tide mark, Dr. Valeriy Skryabin.

####### Description.

**Figure 5.**
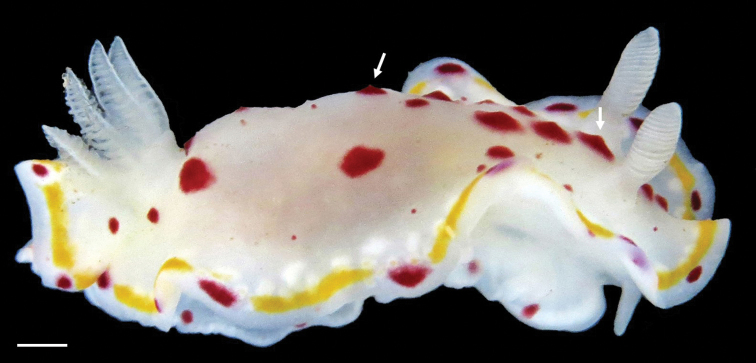
*Goniobranchus* sp. 2 (arrows indicate tubercle-like projections in the dark red/purple spots which give the impression of a conical projection). Photograph Dr. Valeriy Skryabin. Scale bar: 1 mm.

The individual has a white translucent body with dark red / purple spots scattered over the dorsum; a few of the dark red / purple spots have a tubercle-like projection in the middle giving a conical impression (Fig. 5, arrowed). A scattered row of spots extends around the margin of the mantle and the foot. Rhinophores are translucent with white lamellae. Gills are also translucent with a white midrib. The edge of the mantle has a submarginal bright yellow band and an interior ring of opaque white glands.

####### Distribution.

Kuwait (this study).

####### Remarks.

The individual recorded has a submarginal ring of translucent white glands just inside the prominent bright orange band similar to *G. tumuliferus* (Collingwood, 1881; see also Gosliner et al. 2015: 229). However, in *G. tumuliferus* the rhinophores and tentacles have opaque white tips (Gosliner et al. 2015; Mehrotra et al. 2021), which was not observed in the individual recorded during this study. The translucent white glands with dark red / purple spots interrupting the bright orange band is a character combination of what has been observed in *Goniobranchus kitae* Gosliner, 1994 from Madagascar and *G. bimaensis* (Bergh, 1905) from the Indo-West Pacific. Probably an undescribed species.

##### Genus *Hypselodoris* Stimpson, 1855

###### Hypselodoris infucata

Taxon classificationAnimaliaNudibranchiaChromodorididae

(Rüppell & Leuckart, 1830)

B4FF8D26-3C09-544C-BF09-A1C2619F852D

Figure 6A, B

Doris infucata Rüppell & Leuckart, 1828–1830: tab X, 34, fig. 3 (northern African Red Sea).

####### Photographic record.

SAASC, Al Khiran, 2 July 2013, two individuals photographed at 3 m depth on a rock culvert, R. Dinesh Kumar.

####### Description.

**Figure 6.**
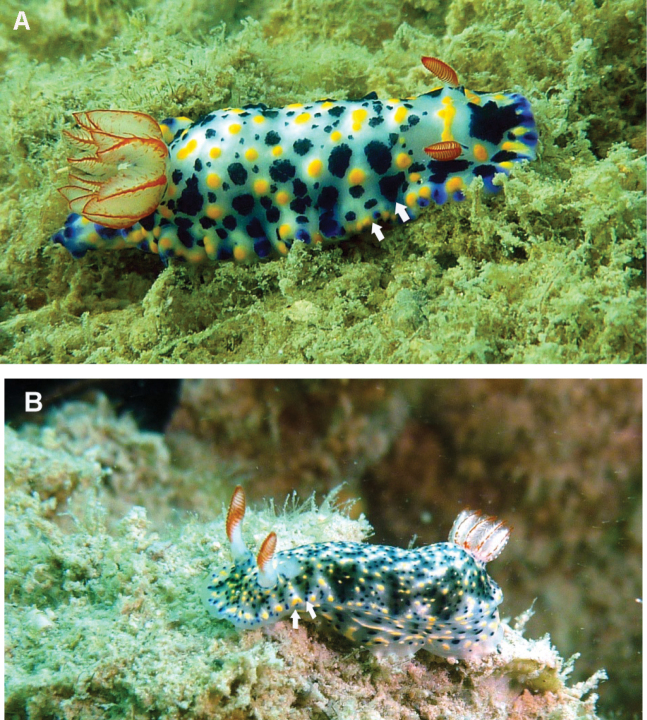
**A***Hypselodoris infucata* (Rüppell & Leuckart, 1830) **B** colour morph. Arrows indicate alternate dark blue-green and pale blue triangles, a diagnostic feature of this species. Photograph R. Diniesh Kumar.

The two individuals in the photographs have a slender white body with blue, yellow, and black spots scattered all over. At the mantle margin, triangular dark blue-green and pale blue patches alternate (Fig. 6A), and bright yellow spots are scattered on both the dorsum and foot. The rhinophores are bright orange-red and the core is white. The oral tentacles are bright orange-red at the midrib and tip (Fig. 6A). A row of prominent dark blue blotches occurs on the either side at the edge of dorsum.

####### Distribution.

Indo-West Pacific species and a Lessepsian migrant (Rudman 1977; Yonow 2008), Oman, South Africa, Philippines, Australia (Debelius 1996), Madagascar, Bali, Indonesia, Papua New Guinea, and Hawaii (Johnson and Valdés 2001; Gosliner et al. 2008), Gulf of Kutch and Lakshadweep, India (Apte 2009; Apte et al. 2010), Larak and Lavan islands, Iran (Rezai et al. 2016), Mozambique (Tibiriçá et al. 2017), Pakistan (Gul, 2019), Thailand (Mehrotra et al. 2021), and Kuwait (this study).

####### Remarks.

This species exhibits a high degree of variability in colour pattern and the bright yellow spots observed in the individual during the present study was similar to a specimen recorded from Eilat, northern Red Sea (Ben Tov 2003). A second colour morph (Fig. 6B) was also recorded with triangular blue grey patches on the either side of the dorsum as illustrated in Yonow (2008). *Hypselodoris infucata* can be easily confused with *H. kanga* Rudman, 1977 due to morphological similarities (Rudman 2007; Mehrotra et al. 2021). In *H. infucata* the gills are rather simple with a bright red line on the outer and inner edges, whereas in *H. kanga*, they are triangular with three lines and, distinctively, with white or yellow spots in-between (Rudman 2007). Bluish purple lines usually occur in the dorsum of *H. kanga* (Mehrotra et al. 2021); however, individuals observed in this study only have dark blue or black spots. *Hypselodoris infucata* differs externally from another congener, *H. roo* Gosliner & Johnson in Epstein et al. 2018, in not having a white spot at the base of the rhinophores on the posterior side and a broad posterior portion of the notum (Epstein et al. 2018). A new record to Kuwait.

###### Hypselodoris

Taxon classificationAnimaliaNudibranchiaChromodorididae

sp.

9E424663-88C1-5E54-A4D2-BAFC349DBF82

Figure 7

####### Photographic record.

SAASC, Al-Khiran, 23 March 2013, one individual on an unidentified sponge photographed at 3.5 m depth, R. Dinesh Kumar.

####### Description.

**Figure 7.**
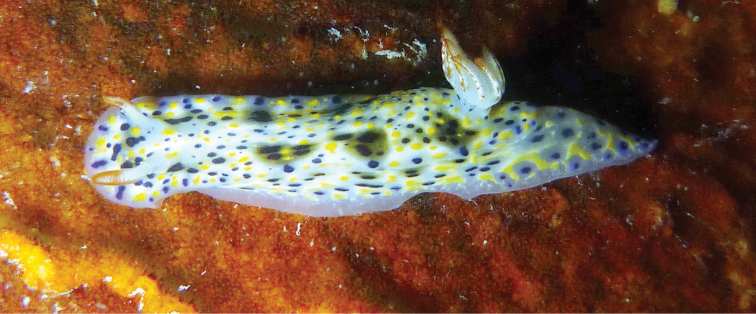
*Hypselodoris* sp. on an unidentified sponge. Photograph R. Dinesh Kumar.

The individual photographed has a bluish grey body with yellow and black spots. The margin of the mantle is thin; yellow and black spots extend onto the foot. A prominent row of black blotches is present on the either side of the dorsum. Rhinophores are tipped red-orange, with a translucent white base (Fig. 7). Gills are orange-red at the tips and the midribs are interrupted with white bands. A circular row of blue spots extends onto the base of the slightly elevated gill pocket.

####### Distribution.

Kuwait (this study).

####### Remarks.

The individual in this study has similarities in colour pattern with two recently described species, *H. confetti* (Johnson & Gosliner in Epstein et al. 2018) and *H. roo*. In *H. confetti*, the gills have purple lines and red-orange tips and in *H. roo* the gills are bright orange-red at tips with two red lines on the exterior side and one on the interior. However, the individual in this study has gills with orange-red midribs and tips (Fig. 7). The bases of the rhinophores are purple in *H. confetti* and red in *H. roo* with a prominent opaque white spot on the posterior side (Epstein et al. 2018), which is clearly absent in the individual recorded in this study as it only has orange-red tipped rhinophores with white bases (Fig. 7). In *H. roo*, the posterior portion of the notum has a tapering shape, which was also observed in this individual (Fig. 7). A new record to Kuwait and the APG.

##### Family Facelinidae Bergh, 1889

**Genus** *Caloria* **Trinchese, 1888**

###### Caloria indica

Taxon classificationAnimaliaNudibranchiaGlaucidae

(Bergh, 1896)

B91146E7-585E-5F81-B3C5-F946FF65CB4A

Figure 8

Learchis indica Bergh, 1896: 385–394, figs 1–4 (Ambon, Indonesia).

####### Photographic record.

SAASC, Al-Khiran, 18 November 2014, one individual on sand and rock mixed substratum photographed at 3.5 m depth, Don Christopher Pereira.

####### Description.

The body is slender, translucent white, with a marking of white lines on the dorsum up to the rhinophores. Orange markings are prominent on the anterior part in front of the cerata and along the sides between the cerata. The rhinophores are smooth, white in colour, orange at the base with a prominent orange band at the middle. The oral tentacles also appear white, long, and slender, with basal orange markings. The cerata are fusiform with white, brown, and blue bands and a translucent white tip. The tail is white, long, and pointed.

####### Distribution.

Indo-West and Central Pacific, Hawaii, Japan, Australia, Indonesia, India, South Africa, Tanzania (Gosliner 1987; Yonow 2008; Gosliner et al. 2015), India (Sreeraj et al. 2012), Maldives (Yonow 1994b), Thailand (Mehrotra et al. 2021), Myanmar (Sanpanich and Duangdee 2019), Papua and New Guinea (Baine and Harasti 2007), Christmas Island, Fiji, New Caledonia, Oman, Seychelles, and Solomon Islands (Gosliner et al. 2008), and now Kuwait (this study).

####### Remarks.

Inhabits coral reef areas (Mehrotra et al. 2021). Feeds on hydroids (Yonow 2008; Gosliner et al. 2015). A new record to Kuwait and the APG.

**Figure 8.**
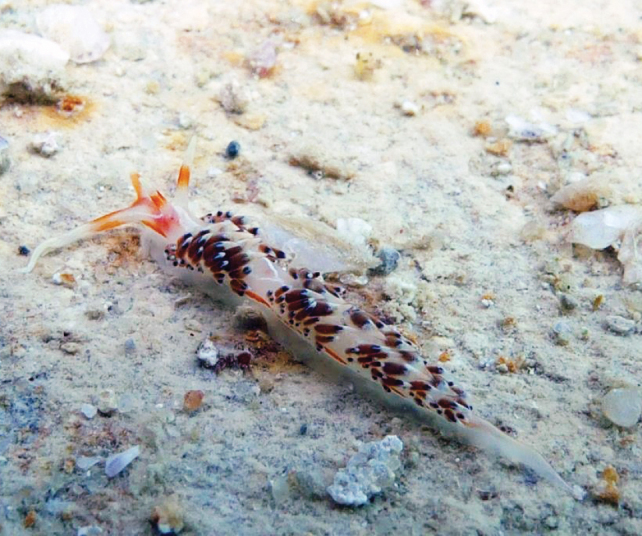
*Caloria indica* (Bergh, 1896). Photograph Don Christopher Pereira.

## Discussion

Kuwait’s rich and unique marine biodiversity is poorly documented and more co-ordinated biodiversity assessments for sustainable management are essential (e.g., Edmonds et al. 2021). The offshore and inshore coral reef habitats are important for several invertebrate and vertebrate species (Papathanasopoulou and Zogaris 2015). Post-gulf war economic development of Kuwait has led to innovative coastal development projects (e.g., Sabah Al Ahmad Sea City) which has increased the extent of both the shoreline and coastal habitats, enhancing marine biodiversity and fisheries (Jones and Nithyanandan 2012b; Nithyanandan 2012; Myers and Nithyanandan 2016; Nithyanandan et al. 2016).

In this study five new records of heterobranch sea slugs to Kuwait and the APG and two potentially new species are reported. Furthermore, this study increases the total number of heterobranch fauna of Kuwait to 40 species, which is 28% of the number reported from both the APG and Gulf of Oman (Amini-Yekta and Dekker 2021).

The occurrence of diverse habitats such as sand, mud flats, rocks, coral reefs, seagrass beds, etc. provides many more opportunities to examine and expand knowledge of the heterobranch diversity in Kuwaiti waters. Harsh environmental conditions in the APG waters of Kuwait potentially governs the impoverished biodiversity of marine biota. Extensive coastal development in the Arabian Peninsula with wide range of artificial marine substrates such as breakwaters, jetties etc. serves as viable benthic habitats attracting colonisation of marine biota (Burt and Bartholomew 2019). In Kuwait, availability of a wide range of benthic substrates both in natural habitats (Al-Kandari et al. 2020) and massive coastal development such as SAASC (Jones and Nithyanandan 2012a) serve as important areas for nudibranch diversity. The colonisation of benthic invertebrates such as sponges, hydroids, etc. in SAASC (Jones and Nithyanandan 2012a; Nithyanandan 2012), which are key prey items for sea slugs (McDonald and Nybakken 1997), could potentially attract them to these artificial habitats. A recent intensive study on sea slug diversity from Thailand indicates the importance of understanding habitat diversity (both natural and artificial) and ecology which drives the functional diversity (Mehrotra et al. 2021).

Historically in Kuwaiti waters efforts were only laid to understand the diversity of heterobranchs (Glayzer et al. 1984; Jones 1986; Al-Yamani et al. 2012; Nithyanandan 2012). Thus, a huge knowledge gap exists in understanding habitat diversity, food preferences, predator-prey interactions, and animal associations in heterobranchs from this marginal environment, which are key factors in driving its diversity and ecology. In the near future, intensive surveys and collecting efforts should incorporate these objectives which could not only help in documenting the diversity of heterobranchs in Kuwait and in the rest of the Arabian / Persian Gulf but also their valuable ecological functions.

## Supplementary Material

XML Treatment for Chelidonura livida

XML Treatment for Goniobranchus bombayanus

XML Treatment for Goniobranchus

XML Treatment for Goniobranchus

XML Treatment for Hypselodoris infucata

XML Treatment for Hypselodoris

XML Treatment for Caloria indica
